# Joint statement on EPA proposed rule and public availability of data (2019)

**DOI:** 10.1371/journal.pmed.1003014

**Published:** 2019-11-26

**Authors:** H. Holden Thorp, Magdalena Skipper, Veronique Kiermer, May Berenbaum, Deborah Sweet, Richard Horton

**Affiliations:** 1 Editor-in-Chief, *Science* family of journals, Washington, District of Columbia, United States of America; 2 Editor-in-Chief, *Nature*, London, United Kingdom; 3 Executive Editor, *Public Library of Science* (*PLOS*) Journals, San Francisco, California, United States of America; 4 Editor-in-Chief, *Proceedings of the National Academy of Sciences (PNAS) of the United States of America*, Washington, District of Columbia, United States of America; 5 Vice President of Editorial, Cell Press, Cambridge, Massachusetts, United States of America; 6 Editor-in-Chief, The Lancet, London, United Kingdom

Eighteen months after articulating our concerns [1] regarding the 2018 “Strengthening Transparency in Regulatory Science” rule proposed by the Environmental Protection Agency (EPA) [2], we have become more concerned in response to recent media coverage and a 13 November hearing on the role of science in decision-making at the EPA. These events suggest that the proposed rule is now moving toward implementation; whether it includes amendments sufficient to address the concerns raised by us and many others remains a question.

Our previous statement on the proposed rule, authored and published by the editors-in-chief of five major scientific journals in May 2018, reflected alarm that the proposal’s push for “transparency” would be used as a mechanism for suppressing the use of relevant scientific evidence in policy-making, including public health regulations. After the public comment period for the proposed rule closed, the EPA reported more than 590,000 comments from individuals and scientific, medical, and legal groups, many of which articulated similar concerns [3].

As leaders of peer-reviewed journals, we support open sharing of research data, but we also recognize the validity of scientific studies that, for confidentiality reasons, cannot indiscriminately share absolutely all data. Datasets featuring personal identifiers—including studies evaluating genomes of thousands of people to characterize medically relevant genetic variants—are but one example. Such data may be critical to developing new drugs or diagnostic tools but cannot be shared openly; even anonymized personal data can be subject to re-identification, and it has been a longstanding practice for agencies and journals to acknowledge the value of data privacy adjustments. The principles of careful data management, as they inform medicine, are just as applicable to data regarding environmental influences on public health. Discounting evidence from the decision-making process on the basis that some data is confidential runs counter to the EPA stated mission "to reduce environmental risks…based on the best available scientific information” [4].

We are also concerned about how the agency plans to consider options related to existing regulations. Even if a new standard is not applied retroactively, the standard could apply when a regulation is updated; thus, foundational science from years past—research on air quality and asthma, for example, or water quality and human health—could be deemed by the EPA to be insufficient for informing our most significant public health issues. That would be a catastrophe.

We urge the EPA to continue to adopt an approach that ensures the data used in decision-making are the best available, which will at times require consideration of peer-reviewed scientific data, not all of which may be open to all members of the public. The most relevant science, vetted through peer review, should inform public policy. Anything less will harm decision-making that claims to protect our health.

We hope that in the end, decisions that are made to inform the proposed EPA rule will rise above any form of politics, focusing on what’s best for our communities. We encourage anyone with concerns or opinions about this issue to express their views through relevant legislative channels. Whether submitting public comments to the EPA or communicating with lawmakers in Congress, it is important to emphasize that decision-making that affects us all should be informed by nothing less than the full suite of relevant science vetted through peer review.
